# Development of Phosphatized Calcium Carbonate Biominerals as Bioactive Bone Graft Substitute Materials, Part I: Incorporation of Magnesium and Strontium Ions

**DOI:** 10.3390/jfb9040069

**Published:** 2018-12-02

**Authors:** Ingo Sethmann, Cornelia Luft, Hans-Joachim Kleebe

**Affiliations:** 1 Institute of Applied Geosciences, Technische Universität Darmstadt, 64287 Darmstadt, Germany; coluft@students.uni-mainz.de (C.L.); kleebe@geo.tu-darmstadt.de (H.-J.K.); 2 Institute of Geosciences, Johannes Gutenberg-Universität Mainz, 55128 Mainz, Germany

**Keywords:** bone graft substitute materials, porous calcium phosphate, coralline hydroxyapatite, phosphatized sea urchin spines, resorbable implant materials, bioactive implant materials, magnesium, strontium

## Abstract

Synthetic materials based on calcium phosphate (CaP) are frequently used as bone graft substitutes when natural bone grafts are not available or not suitable. Chemical similarity to bone guarantees the biocompatibility of synthetic CaP materials, whereas macroporosity enables their integration into the natural bone tissue. To restore optimum mechanical performance after the grafting procedure, gradual resorption of CaP implants and simultaneous replacement by natural bone is desirable. Mg and Sr ions released from implants support osteointegration by stimulating bone formation. Furthermore, Sr ions counteract osteoporotic bone loss and reduce the probability of related fractures. The present study aimed at developing porous Ca carbonate biominerals into novel CaP-based, bioactive bone implant materials. Macroporous Ca carbonate biominerals, specifically skeletons of corals (aragonite) and sea urchins (Mg-substituted calcite), were hydrothermally converted into pseudomorphic CaP materials with their natural porosity preserved. Sr ions were introduced to the mineral replacement reactions by temporarily stabilizing them in the hydrothermal phosphate solutions as Sr-EDTA complexes. In this reaction system, Na, Mg, and Sr ions favored the formation of correspondingly substituted β-tricalcium phosphate over hydroxyapatite. Upon dissolution, the incorporated functional ions became released, endowing these CaP materials with bioactive and potentially osteoporotic properties.

## 1. Introduction

Annually, more than two million bone graft procedures are performed worldwide [1]. The main objective is the repair of bone defects caused by trauma or tumor resection. Although considered to be the ideal implant material, autografts of cancellous bone are sometimes not available in sufficient quantity, and harvesting has its own potential complications. Blood loss and additional pain, as well as the risk of infection and donor site instability, may retard patient recovery [2,3,4,5,6].

To avoid the drawbacks of autografts, there is a critical need for biocompatible materials for bone graft substitutions with sufficient mechanical strength that become resorbed through the natural bone remodeling process after fulfilling their initial function of bridging and stabilizing the defect [7]. Synthetic materials that most closely resemble the properties and composition of human bone are based on Ca phosphate (CaP) [8,9]. The mineral component of bone consists of hydroxyapatite that contains impurities, according to the generalized chemical formula Ca_5_(PO_4_,CO_3_)_3_(OH,F,Cl,CO_3_) with variable contents [10]. Additionally, bone contains 0.5 wt % Na, 0.25 wt % Mg, and 0.02 wt % Sr (among others) as impurities [11,12] that substitute for Ca.

CaP materials generally show excellent biocompatibility, osteoconductive properties, and the capability to bond directly to newly formed bone tissue [13,14]. Although similar to the mineral content of human bone, pure hydroxyapatite (HA, Ca_5_(PO_4_)_3_OH) implants give unsatisfactory results in clinical applications because they show no osteoinductive ability [15,16,17,18,19]. Whereas pure HA implant materials are capable of forming direct interfacial bonds with the host tissue [20], they are only very slowly degradable, thus impeding resorption through the natural bone remodeling process [21]. In a comparative study, pure β-tricalcium phosphate (β-TCP, Ca_3_(PO_4_)_2_) combined with bone marrow aspirate gave better results than HA material, and its performance came closest to autografts [18]. However, β-TCP resorption is rather too fast, leading to mechanical instability of the implant site [22,23]. As β-TCP is far more resorbable than HA [24], degradation rates of biphasic mixtures can be adjusted to the rate of new bone formation [25]. Biphasic Ca phosphate (BCP) materials offer good osteoconduction and osteointegration, as proven by bone formation within pores after a few months [26,27,28]. Bone regeneration is favored by interconnected macropores, which also promotes the biological fixation of the implant [29,30,31,32,33,34,35,36]. Ultraporous BCP scaffolds with macro-, meso-, and microporosity have shown particularly good results, attracting cells and nutrients by capillary force [37].

Besides porous Ca phosphate bioceramics [9] and bioglass [38,39], alternative materials are based on bovine bone [40,41] or derived from coral skeletons [42] (originally consisting of aragonite, CaCO_3_), which exhibit natural macroporosities. Coral-derived materials are prepared by conversion of the original Ca carbonate materials into HA via pseudomorphic mineral replacement in a hydrothermal phosphate solution [43,44], a process that preserves the macroporosity of the original materials. Similar to bone, these coralline HA products contain minor impurities of Sr and Mg ions, as well as carbonate [45] inherent to the precursor materials. However, due to the lack of resorbability of these HA scaffolds, their applicability as bone graft substitutes is limited until further developments improve the currently existing materials [46]. The solubility of HA can be modified through incorporation of impurity ions [47]. In HA, virtually all ions—Ca as well as phosphate and hydroxyl ions—can be substituted. Carbonate may substitute for phosphate or hydroxyl ions [48]. Incorporation of Mg instead of Ca ions destabilizes the crystal structure of HA: However, this can partly be compensated for by substitution of carbonate ions for phosphate or hydroxyl ions [49]. 

As a promising alternative to coralline HA, sea urchin-derived material (originally consisting of calcite (CaCO_3_), with Ca ions partially substituted by Mg ions) has been tested for bone implants [50]. The phosphatized material consisted mainly of resorbable Mg- and carbonate-substituted β-TCP [50,51,52]. The structure of β-TCP was able to accommodate up to 14–24 mol % Mg ions substituting for Ca ions [53,54]. Therefore, substantial amounts of Mg in the reaction system favored the formation of β-TCP over HA [50,51,52].

Mg has regulatory functions in the biological process of bone mineral formation. A lack of Mg reduces the activity of osteoblasts and osteoclasts and may cease bone growth [55]. Mg-containing Ca phosphate as an implant enhances the adhesion, proliferation, and metabolic activity of osteoblast-like cells [56].

Sr ions stimulate bone formation and inhibit bone resorption, as shown in vitro and in vivo [57,58]. Sr ions promote the formation of new bone by enhancing proliferation of mesenchymal stem cells and osteoblast progenitor cells as well as their differentiation into osteoblasts, which form new bone substance at a higher rate [57,59,60]. Bone resorption is retarded by inhibiting the maturation of osteoclast precursor cells and by inducing apoptosis (cell death) in mature osteoclasts [60,61,62]. In this way, bone mineral density is significantly increased and the risk of osteoporosis-related fractures is decreased [63,64]. Sr-containing bone implants and bone cements can be modified to constantly release Sr ions while they are resorbed [65,66] and thereby act as local anti-osteoporotic drug dispensers. These positive effects of ion substitutions can be combined by co-substitution of different ions [55,65].

The present study was aimed at developing phosphatized Ca carbonate biominerals (PCCB) toward resorbable, bioactive, and antiosteoporotic bone graft substitute materials by the incorporation of Mg and Sr ions. Successfully modified PCCB materials could turn out to be excellent implant materials as an alternative to bioactive CaP ceramics or bioglass by taking advantage of the natural porosity of the starting materials and employing a hydrothermal preparation procedure without the need for high-temperature equipment.

## 2. Results and Discussion

### 2.1. Starting Material

**Figure 1:**
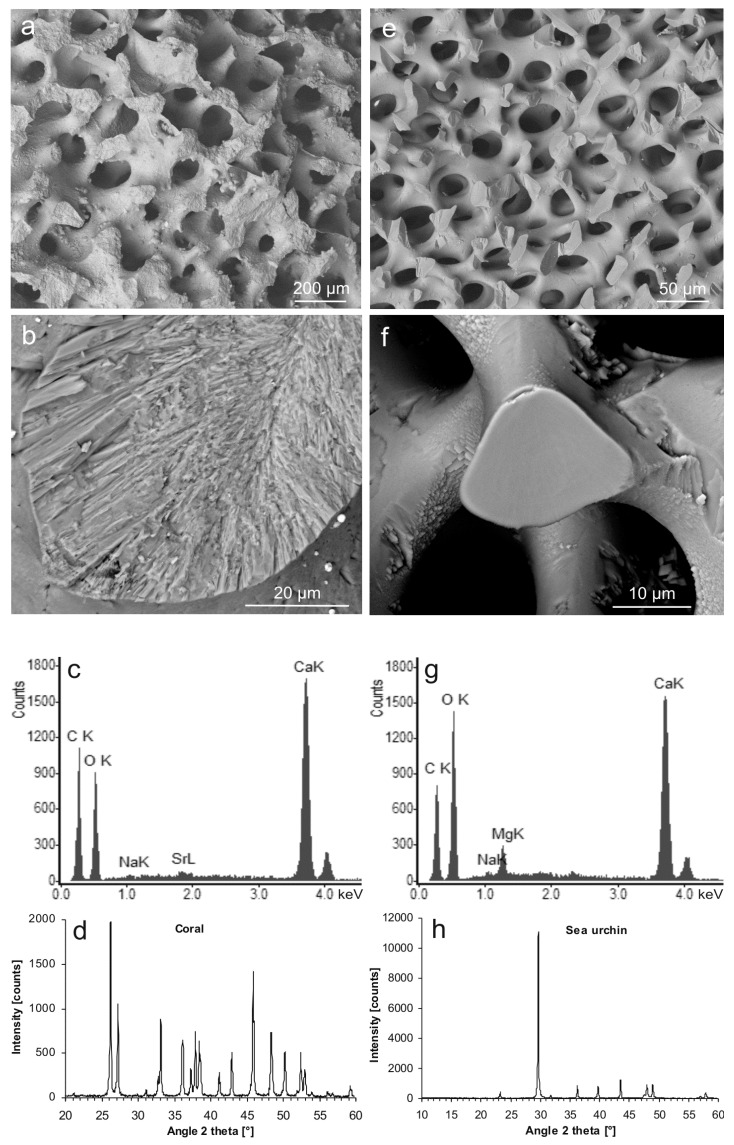
Characterization of the starting materials. (**a**) Porous skeleton of the coral *Porites* sp. (scanning electron microscopy-backscatter electron (SEM-BSE)); (**b**) fracture surface of the coral material revealing the internal microstructure (SEM-BSE); (**c**) chemical composition of the coral skeleton (energy-dispersive X-ray spectroscopy (EDS)); (**d**) XRD pattern of the coral skeleton corresponding to that of aragonite (PDF 00-041-1475) (XRD raw data S1: C14); (**e**) porous material of a spine of the sea urchin *Heterocentrotus mamillatus* (SEM-BSE); (**f**) fracture surface of the sea urchin material showing a massive internal structure (SEM-BSE); (**g**) chemical composition of the sea urchin spine (EDS); (**h**) XRD pattern of the of the sea urchin spine corresponding to that of Mg calcite (PDF 00-043-0697) (XRD raw data S2: SU1).

The starting materials bore interconnected pore systems. In the case of the coral skeleton, the pores were about 100–150 µm in diameter (Figure 1a), and the trabecular material showed a polycrystalline, fibrous internal microstructure (Figure 1b). Chemical and structural characterization by means of energy-dispersive X-ray spectroscopy (EDS) and XRD confirmed the coral material to consist of Ca carbonate (Figure 1c) with the crystal structure of polymorph aragonite (PDF 00-041-1475) (Figure 1d). In comparison, the sea urchin spines contained smaller pores about 20–50 µm in diameter (Figure 1e), and the internal structure of the trabeculae appeared massive in the SEM image (Figure 1f). The material consisted of Mg-bearing Ca carbonate (Figure 1g) with the crystal structure of a polymorph calcite but with a slight shift to larger diffraction angles (i.e., smaller spacing in the crystal lattice) (PDF 00-043-0697) (Figure 1h). From the differences between theoretical massive sample weights (calculated from measured sample dimensions and material densities) and the actual sample weights, the overall porosities were estimated. These calculations were based on aragonite having a density of *d_ara_* = 2.93 g/cm^3^ and on sea urchin calcite having an approximate chemical formula of Ca_0.9_Mg_0.1_CO_3_ [52] and hence a density of *d_Mg-cal_* = 2.75 g/cm^3^ (derived from Althoff [67]). The resulting porosities were about 69.4% ± 0.6% for the coral and about 63.4% ± 1.2% for the sea urchin material.

### 2.2. Hydrothermal Treatment in Pure Water

In a coral sample subjected to hydrothermal treatment in demineralized water at 200 °C for 7 days, the morphology of the sample and the macroporosity were largely preserved (Figure S1a), and the internal microstructure of crystal fibers remained intact (Figure S1b). Phase quantification based on the XRD pattern showed that the original aragonite remained the dominant phase (95 wt %), whereas a replacement reaction produced the calcite polymorph of CaCO_3_ as a minor phase (5 wt %; Figure S1c; XRD raw data S3: C30). The resulting material appeared slightly more brittle than the original coralline aragonite.

Aragonite, as a metastable polymorph of Ca carbonate, was replaced via a dissolution in the hydrothermal water coupled with immediate reprecipitation of the ions as the thermodynamically stable polymorph calcite [68], which involved an increase in molar volume by about 8.0% (disregarding the solubility of calcite in the hydrothermal fluid). Increasing the volume at the reaction front (in a confined space) created stress in the bulk of the material, which may have induced fractures [68] and increased the brittleness of the calcite pseudomorph produced.

In the case of Mg calcite from sea urchin spines, hydrothermal treatment with demineralized water did not change the morphology, the internal microstructure, the mineral phase, or the mechanical properties.

### 2.3. Hydrothermal Phosphatization of Coral Skeletons

**Figure 2:**
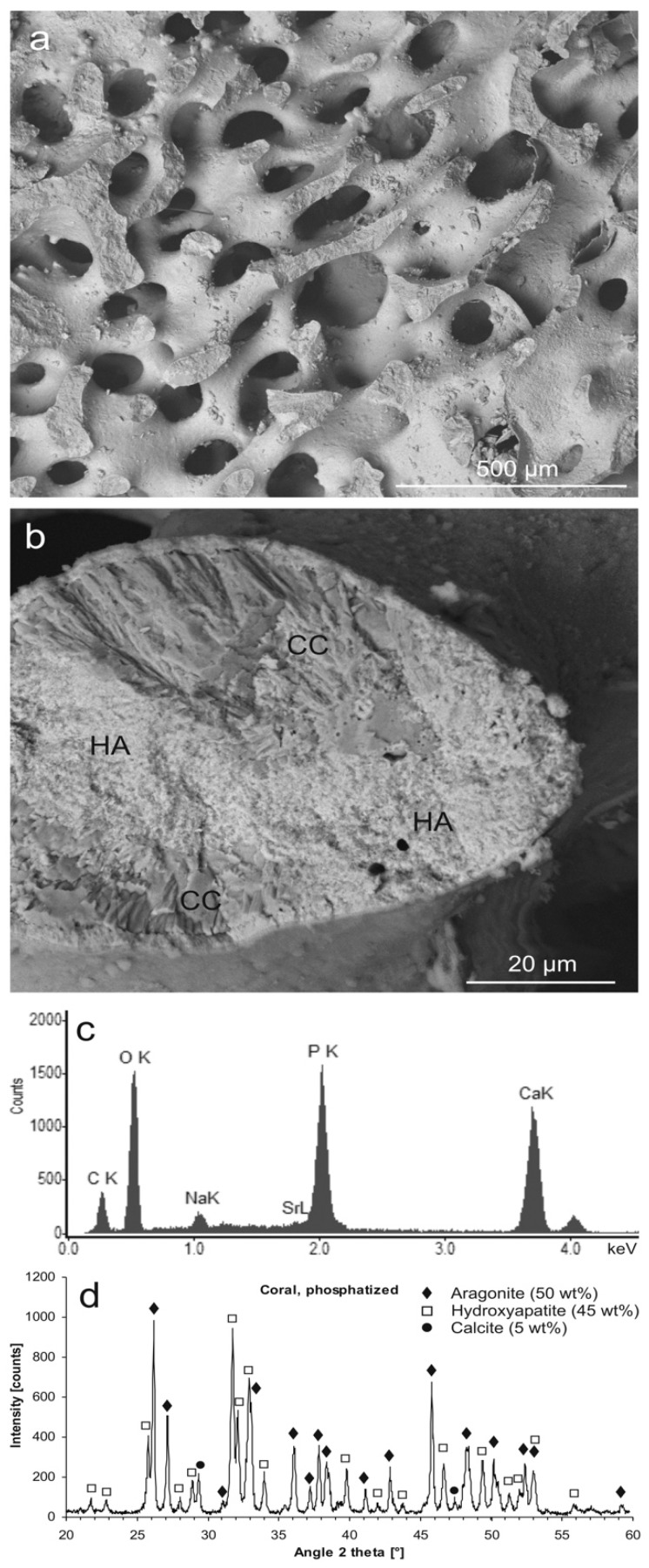
Hydrothermally phosphatized coral skeleton. (**a**) Aragonite skeleton partly converted into pseudomorphic hydroxyapatite (HA) (SEM-BSE); (**b**) a fractured trabecula showing the native, fibrous Ca carbonate microstructure (CC, darker shading), partly converted into microcrystalline HA (lighter shading) (SEM-BSE); (**c**) chemical composition of the phosphatized material (EDS); (**d**) mineral phases contained in the partly phosphatized coral material identified by XRD: aragonite (50 wt %, PDF 00-041-1475), hydroxyapatite (45 wt %, PDF 00-009-0432), calcite (5 wt %, PDF 00-047-1743); XRD raw data S4: C3.

After hydrothermal treatment of coral samples in a phosphate solution, the outer shape of the sample as well as the internal morphology of the pore system was preserved (Figure 2a), whereas the fibrous microstructure was partially changed into a microcrystalline and microporous, but pseudomorphic, material (Figure 2b). The native aragonite of the coral was partly converted into Na- and carbonate-bearing Ca phosphate (Figure 2c) with the crystal structure of HA (Figure 2d). The material consisted of about 50 wt % native aragonite and the newly formed phases of calcite (5 wt %) and HA (45 wt %). After phosphatization, the material was considerably more brittle than the original coralline aragonite.

In hydrothermal phosphate solution, this Ca carbonate phase transition in coral skeletons competed with the formation of HA. This mineral replacement reaction took place via dissolution of aragonite coupled with the reprecipitation of the released Ca ions as Na-substituted and carbonated HA [69], involving a decrease in volume by about 6.9% (disregarding the solubility of the HA in the hydrothermal phosphate solution). The volume reduction allowed for a microporosity to form that enabled the diffusive ion exchange between the bulk solution and the fluid phase at the reaction front that is necessary for the replacement reaction to continue [70]. The reaction occurred according to the following simplified equation:

9 CaCO_3_ + Na^+^ + 5 HPO_4_^2−^ + 3 H_2_O ⇒ Ca_9_Na(PO_4_)_5_(CO_3_)(OH)_2_ + 8 HCO_3_^−^ + OH^−.^ (1)

Phosphatized coral skeletons (also termed coralline HA) as bone graft substitute materials have been found to be biocompatible, osteoconductive, and osteointegrative due to their bone-like chemical composition and their porous structure. However, these HA materials are brittle and have to be regarded as (almost) non-resorbable, permanent implants, which restricts their application to well-contained, non-load-bearing applications [46].

However, conversion of the original coral material from aragonite into HA was only partially achieved under the conditions applied. Upon application as bone graft substitute material, the original aragonite became resorbed faster than HA [71]. Hence, residual aragonite could enhance the resorbability of coralline HA implants [72]. Furthermore, the coralline HA materials produced were found to be more brittle than the original coral materials, presumably due to microstructural alterations and the development of microporosity during conversion. Furthermore, the native coral material contained an organic matrix (about 0.8 wt %) consisting mainly of sulphated polysaccharides intimately associated with the aragonite fibers [73]. The organic matrix probably reduced the brittleness of the native material. During phosphatization by mineral replacement, the organic matrix was assumed to become dissociated from the mineral phase and was removed from the material at least partly. Compressive strengths previously reported for native *Porites* coral skeletons were in the range of 14.1 MPa (dry) and 9.7 MPa (wet), whereas those of completely phosphatized coralline HA were significantly lower at 5.7 MPa (dry) and 2.6 (wet) [74]. The mechanical robustness of coralline HA materials may, therefore, benefit from some residual Ca carbonate as well. However, residual organic contents may increase the risk of immune reactions.

### 2.4. Hydrothermal Phosphatization of Sea Urchin Spines

Initially, it was also intended to incorporate Mg ions into the coral material during the process of phosphatization. For this purpose, Mg ions were supposed to be temporarily stabilized in the phosphate solution by forming complexes with EDTA. However, this approach was not successful because Mg ions could not be stabilized in sufficient amounts in this way.

As an alternative route to producing an Mg-doped CaP material with natural, pervasive porosity through hydrothermal treatment, sea urchin spines consisting of Mg calcite were employed instead of the coral skeleton.

**Figure 3:**
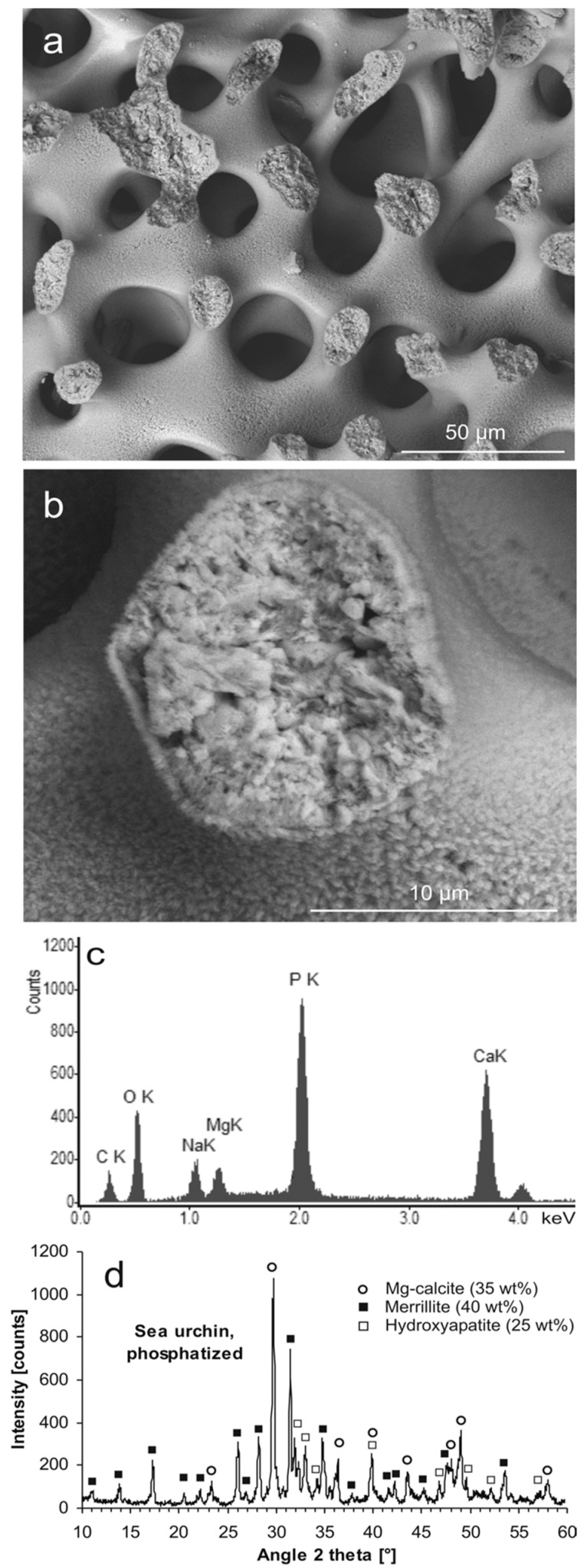
Hydrothermally phosphatized sea urchin spine. (**a**) Mg calcite scaffold partly converted into phosphatic material (SEM-BSE); (**b**) a fractured trabecula showing the microgranular and microporous structure of the pseudomorphic phosphate material (SEM-BSE); (**c**) chemical composition of the Na- and Mg-bearing phosphatized material (EDS); (**d**) mineral phases contained in the partly phosphatized sea urchin material identified by XRD: Mg-calcite (35 wt %, PDF 00-043-0697), merrillite (40 wt %, PDF 01-076-8368), hydroxyapatite (25 wt %, PDF 00-009-0432); XRD raw data S5: SU3.

Hydrothermal phosphatization of sea urchin spines resulted in a pseudomorphic sample (Figure 3a) with natural porosity preserved and a microcrystalline and microporous internal structure of the material (Figure 3b), largely similar to the coral-derived material. However, in contrast to the phosphatized coral material, the phosphatized sea urchin sample contained substantial amounts of Mg (Figure 3c). In this case, the material consisted mainly of the mineral merrillite (Ca_9_NaMg(PO_4_)_7_) (40 wt %), corresponding to PDF 01-076-8368 (structurally similar to β-TCP), combined with minor amounts of HA (25 wt %) and residual Mg calcite (35 wt %) (Figure 3d). During preparation of the phosphatized samples for analysis, it was conspicuous that the sea urchin-derived material was much less brittle than the coral-derived material.

Upon dissolution of Mg calcite in Na phosphate solution and coupled CaP precipitation, Mg ions released together with Ca ions shifted the reaction equilibrium from HA toward a β-TCP type of precipitate: β-TCP could readily accommodate considerable concentrations of Na and Mg ions in its crystal structure, forming the mineral merrillite. The conversion of Mg calcite into merrillite involved a volume reduction by 3.3% (disregarding additional loss of volume due to the solubility of merrillite in the hydrothermal solution). Conversion of Mg calcite into the minor product phase HA involved a decrease in volume by 11.0%. Taking into account the ratio by which merrillite and HA were produced, an effective volume reduction by 6.2% could be calculated for the biphasic product material. Hence, sufficient porosity could form to enable ion transport from the bulk solution to the reaction front and vice versa, allowing the replacement reaction to continue. The replacement of Mg calcite by merrillite occurred according to the following simplified reaction equation:

Ca_9_Mg(CO_3_)_10_ + Na^+^ + 7 HPO_4_^2−^ + 3 H_2_O ⇒ Ca_9_NaMg(PO_4_)_7_ + 10 HCO_3_^−^ + 3 OH^−.^ (2)

When applied as bone graft substitutes, similar sea urchin-derived materials were found to be biocompatible, and new bone was formed inside the pores progressively from the outside to the center of the implant [48]. Due to the expected resorbability of sea urchin-derived biphasic scaffolds of merrillite and HA, the minimum pore size of 100 µm, recommended for bone replacement materials, was not necessarily a critical limitation here [35].

Compared to the coral-based material, the material based on sea urchin spines of the species *Heterocentrotus mamillatus* were much less brittle and, in previous reports, scored considerably higher in compressive strength, with 42–49 MPa for the native Mg calcite (dry) [50,75] and 23 MPa for completely phosphatized material (dry) [50]. Sea urchin calcite contains less than 0.1 wt % organic substances (glycoproteins), which are intimately associated with the mineral phase as well [76]. This organic matrix induces a certain flexibility of the otherwise brittle calcite material. Here as well, the phosphatization by hydrothermal mineral replacement dislocated the organic matrix from the mineral phase and probably removed it from the material at least partly. The reduced strength of the phosphatized material was probably caused by the microcrystalline internal structure and the microporosity of the pseudomorphic material produced together with the loss of the strengthening effect of the organic matrix. Here, again, an incomplete conversion of Mg calcite into phosphate materials may preserve a higher compressive strength, which would be advantageous for an implant material. Although a risk of immune reactions due to the presence of residual organic matrix cannot be excluded, the concentration of these remains is expected to be very low.

Most previous studies utilized hydrothermal solutions of diammonium hydrogen phosphate to accomplish phosphatization. However, upon phosphatization of Mg-bearing carbonate minerals in ammonium-containing solutions, the formation of large, platelet-shaped crystals of dittmarite ((NH_4_)Mg(PO_4_)_3_OH) as a minor phase was discovered [77]. For avoiding potentially adverse effects of such crystals on the mechanical properties of the sea urchin-derived materials, the use of hydrothermal solutions of disodium hydrogen phosphate, inducing the formation of microcrystalline merrillite, could be advantageous.

### 2.5. Modification with Strontium Ions

To effect an incorporation of Sr ions into the material during its hydrothermal conversion in phosphate solution, these solutions were prepared with Sr ions as dissolved Sr-EDTA complexes, which prevented the immediate precipitation of an Sr phosphate phase.

**Figure 4:**
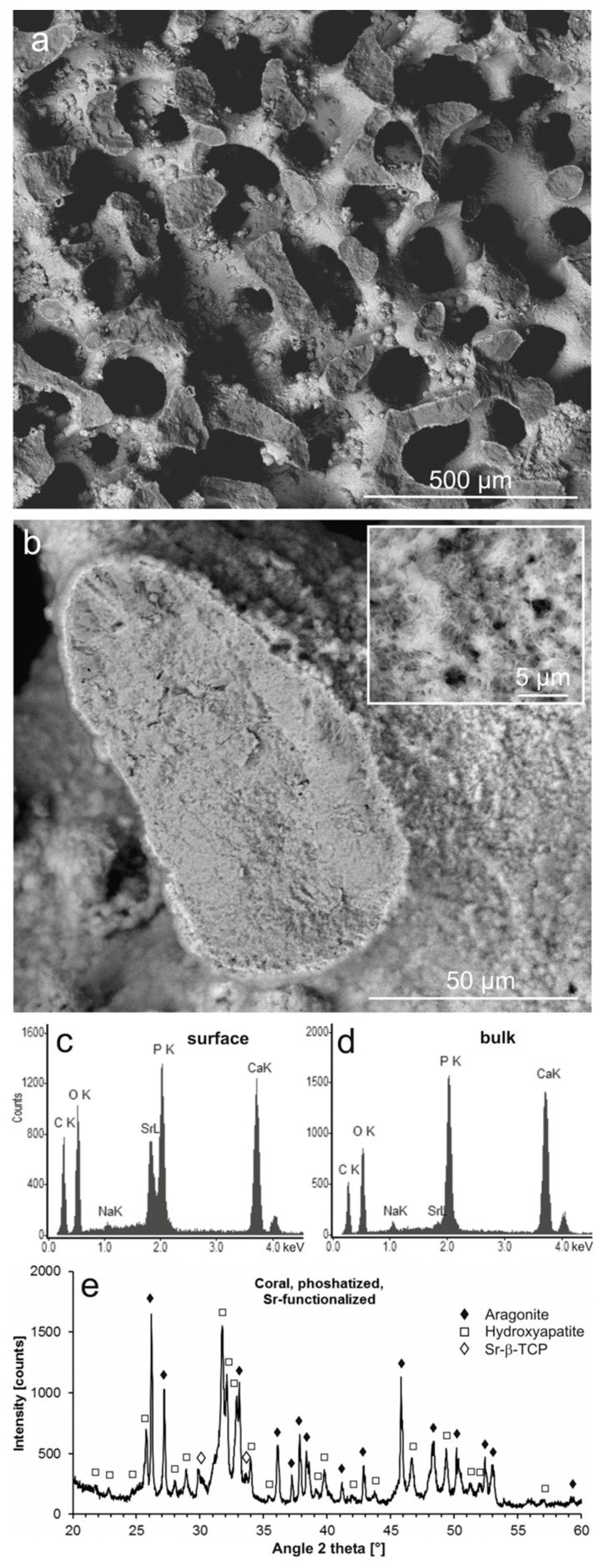
Phosphatized and Sr-modified coral skeleton. (**a**) Aragonite skeleton partly converted into pseudomorphic phosphate material (SEM-BSE); (**b**) a fractured trabecula showing the microcrystalline structure of the phosphate material in the interior (darker shading) and a surface layer of small crystals (see inset) with heavier elements (lighter shading) (SEM-BSE); (**c**) chemical composition of the surface material (EDS); (**d**) chemical composition of the phosphatized bulk material (EDS); (**e**) crystalline phases contained in the partly phosphatized and Sr-modified coral material, identified using XRD (aragonite (PDF 00-041-1475), hydroxyapatite (PDF 00-009-0432), Sr-substituted β-TCP [78]); XRD raw data S6: C32.

Similar to the phosphatization without Sr ions, the conversion produced a pseudomorph of the original aragonitic coral skeleton (Figure 4a), with a microcrystalline internal structure (Figure 4b). However, as an additional feature, the surface was covered with a thin layer of aggregated, platelet-shaped microcrystals with significantly higher density than the bulk material, as can be seen in the scanning electron microscopy-backscatter electron (SEM-BSE) images (Figure 4b with inset). EDS confirmed the surface layer to consist of an Sr-CaP with a high Sr content (Figure 4c), whereas the bulk material was composed of CaP with only small amounts of Na and Sr incorporated (Figure 4d). According to an XRD analysis, the material consisted of residual aragonite and pseudomorphic HA as the dominant phases, combined with a minor phase of Sr-substituted β-TCP [78] ((Ca,Sr)_3_(PO_4_)_2_) (Figure 4e), which presumably formed the surface layer. The Sr content of Sr-β-TCP may vary, but based on the position of the (0.2.10) reflex of Sr-β-TCP at 29.84° 2θ, the maximum degree of substitution of Sr ions for Ca ions in the β-TCP crystal lattice could be estimated to exceed 60% (cf. Bigi et al. [78]). 

**Figure 5:**
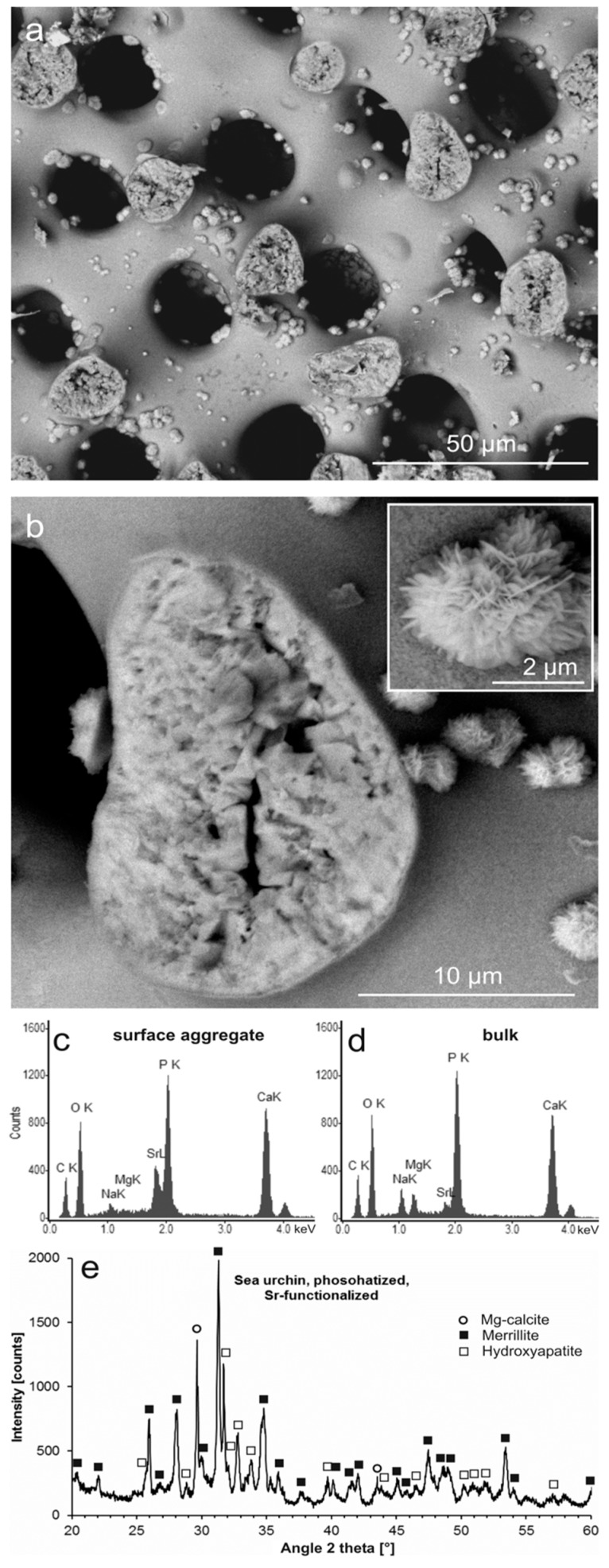
Phosphatized and Sr-modified sea urchin spine. (**a**) Mg calcite scaffold partly converted into pseudomorphic phosphate material (SEM-BSE); (**b**) a fractured trabecula showing the microcrystalline and microporous structure of the phosphate material in the interior (darker shading) and rose-like aggregates of phosphate crystals containing heavier elements (lighter shading) grown on the surface (see inset) (SEM-BSE); (**c**) chemical composition of a rose-shaped crystal aggregate on the material surface (EDS); (**d**) chemical composition of the phosphatized bulk material (EDS); (**e**) mineral phases contained in the partly phosphatized and Sr-modified sea urchin material, identified by XRD (Mg-calcite (PDF 00-043-0697), merrillite (PDF 01-076-8368), hydroxyapatite (PDF 00-009-0432)); XRD raw data S7: SU24.

Sea urchin Mg calcite was pseudomorphically replaced in Sr-EDTA-containing phosphate solution as well (Figure 5a). The interior of the material was microcrystalline with a secondary microporosity, whereas the surfaces were smooth but studded with rose-shaped aggregates of small, platelet-shaped crystals (Figure 5a,b). EDS analysis revealed the rose-shaped surface aggregates to consist of Sr-rich CaP containing low concentrations of Na (Figure 5c). Corresponding analysis of the pseudomorphic CaP of the bulk material demonstrated a different composition with only low concentrations of Sr ions, but with substantial amounts of Na and Mg ions (Figure 5d). Identification of crystalline phases via XRD yielded merrillite as the dominant phase, considerably less but still significant amounts of HA, and a minor residue of the original Mg calcite (Figure 5e). The rose-shaped aggregates presumably consisted of Sr-β-TCP and represented structures analogous to the surface layers of the corresponding coral material (Figure 4). However, no Sr-β-TCP was identified in Figure 5e, probably due to its structural similarity to the dominant merrillite phase and the relatively small amount of Sr-rich, rose-shaped aggregates.

For incorporation of Sr ions into the materials during phosphatization, these ions had to be stabilized in the hydrothermal phosphate solutions as Sr-EDTA complexes to become reagents in the mineral replacement reaction. EDTA complex stabilities differ for different cations: Ca^2+^ > Mg^2+^ > Sr^2+^ >> Na^+^ [79]. At the surface of the dissolving Ca carbonate scaffolds, it was hence to be expected that released Ca ions competed with Sr ions for EDTA complexation sites, causing the release of Sr ions. Additionally, Sr ions were presumably released from EDTA due to other causes: (i) Hydrogen phosphate ions in solution competed directly with EDTA for binding Sr ions, (ii) Sr-EDTA as a polycarboxylate adsorbed on Ca carbonate surfaces [80], and (iii) EDTA became thermally decomposed [81].

These processes of transport and release of Sr ions by EDTA led to their precipitation with phosphate ions, mostly not as Sr-substituted HA, but as Sr-β-TCP at the surfaces of the materials (Figure 4 and Figure 5). A portion of the Sr ions, however, remained mobile throughout the entire reaction time or became remobilized and transported through the newly formed micropores to precipitate at the reaction front, whereas mineral replacement advanced toward the bulk of the material. 

Sr-modified coralline HA prepared by hydrothermal mineral replacement was already described in an earlier study [82]. However, in contrast to our approach of simultaneous phosphatization and Sr modification in a single preparation step, the former protocol required a separate hydrothermal treatment for Sr modification subsequent to the phosphatization. Unfortunately, the distribution of Sr in the material was not recorded in that study.

### 2.6. Dissolution and Release of Ions

The benefits of Mg and Sr ions as constituents of bone graft substitute materials came into effect upon their dissolution in the body fluid after the implant surgery. As an experimental approach to this process, phosphatized and Sr-modified samples of coral and sea urchin material were held in Ringer’s solution at body temperature. The solutions resulting from these experiments as well as a sample of the pristine solvent (sample Ringer) were analyzed using atomic absorption spectroscopy (AAS) (Table 1).

The concentrations of dissolved Mg ions were generally low, between 0.1 and 0.2 mg/L. Coral-derived samples released (almost) no Mg ions, as became clear in comparison to the corresponding control measurement for impurities in pristine Ringer’s solution. The sea urchin-derived material, however, clearly released some additional Mg ions into the solution. The measured concentrations of Sr ions in solution were considerably higher for both sample types, with the Sr concentrations of Sr ions released from the coral-derived material being four times as high compared to the sea urchin-derived material. In the pristine solvent, concentrations of Sr ions were below the detection limit. In general, the concentrations of the ions determined hardly showed any discernible increase during the reaction time between 24 h and 72 h in Ringer’s solution, presumably indicating that the solutions became nearly saturated with respect to the relevant substances within 24 h. 

The incorporation of Mg and Sr ions into the materials was intended as a functionalization for bioactivity and antibacterial effect upon implantation. For sea urchin-derived materials, the concentrations of Mg ions released upon dissolution in Ringer’s solution were within a range previously found to be advantageous for osteoblast-like cell proliferation [83]. For both coral and sea urchin-derived materials, the concentrations of the released Sr ions were on the low side of the range beneficial for proliferation of mesenchymal stem cells and osteogenic differentiation [66]. A combination of Mg and Sr ions released into the solution, as in the case of our sea urchin material, reduces the respective concentrations necessary for a beneficial effect on cell proliferation [65]. Therefore, the biomineral-based materials produced here hold potential for an application as bioactive bone graft substitute materials. These new materials may be on a par with existing fully synthetic materials, such as CaP ceramics or bioglasses, and could turn out as worthwhile alternatives. The actual performance of these modified PCCB materials in an implant situation remains, however, to be tested.

## 3. Materials and Methods

### 3.1. Starting Materials

Two different Ca carbonate biomineral structures with open porosity were chosen as starting materials for this study, a coral skeleton and sea urchin spines. In the first case, the basal skeleton of the scleractinian coral *Porites* sp. was used. The material was cut to produce rectangular samples with sizes of about 0.6–0.8 cm^3^ and weights of about 0.5–0.7 g. In the second case, spines of the sea urchin *Heterocentrotus mamillatus* were cut into discs about 10.2–10.6 mm in diameter and 5.6–6.1 mm in thickness (about 0.5 cm^3^ and 0.5 g). Subsequently, all samples were ultrasonically cleaned in deionized water to remove debris from the pores and, after drying on tissues, held in hydrogen peroxide solution (H_2_O_2_ 10%) for three days to remove organic material from the mineral surfaces. Afterwards, the samples were thoroughly rinsed with deionized water and dried on tissues. Samples were weighed, and their individual dimensions were measured using a micrometer gauge. The porosities of the samples were estimated from the differences between their actual weights and the weights calculated for the samples assuming massive materials.

### 3.2. Hydrothermal Treatment

In a basic set of experiments, samples were subjected to hydrothermal treatment with either pure deionized water or solutions of Na_2_HPO_4_ · 2 H_2_O (1.0 mol/L) prepared with deionized water preheated to 60 °C. For phosphatization of the materials, each sample was placed in 35 mL solution in a Teflon^TM^-lined autoclave (steel bomb, 125 mL volume; Parr Instrument, Frankfurt, Germany) preheated to 60 °C and then held at 200 °C for 168 h.

Additional experiments were conducted with modified hydrothermal solutions to implement Sr ion incorporation into the material simultaneously to the phosphatization process. These solutions were prepared by first dissolving ethylenediaminetetraacetic acid disodium salt dihydrate (Na_2_EDTA·2 H_2_O; 0.2 mol/L) in deionized water preheated to 60 °C, followed by SrCl_2_·6 H_2_O (0.1 mol/L) and stirring for 10 min before Na_2_HPO_4_·2 H_2_O (1.0 mol/L) was slowly added. The solution was clear without any precipitate or turbidity when poured into the preheated autoclaves together with the samples (35 mL solution and 1 sample per autoclave). Similarly to the basic experiments, samples were held in this Sr-containing solution at 200 °C for 168 h.

After cooling down to about 60 °C, the resulting samples were extracted from the solution, thoroughly washed in deionized water, and dried on a tissue that absorbed the fluid from the pores to avoid precipitation by evaporation as much as possible.

### 3.3. Sample Characterization

Samples were cleaved with a steel blade to expose fresh fracture surfaces of the material for characterization with scanning electron microscopy (SEM; FEI ESEM Quanta 200 FEG, ThermoFisher Scientific, Hillsboro, OR, USA) at 12.5 kV accelerating voltage for imaging their morphology and material contrasts in backscatter electron (BSE) imaging mode. Additionally, SEM-integrated energy-dispersive X-ray spectroscopy (EDS; EDAX, Mahwah, NJ, USA) was applied for determining the chemical compositions of the sample materials. For these applications, carbon tabs were used to mount cleaved samples onto aluminum stubs before, in most cases, samples were carbon-coated for electron conductivity. Here, the SEM was operated in high-vacuum mode. 

Selected samples were ground to powder in an agate mortar prior to identification of the crystalline phases by X-ray diffraction (XRD; Siemens D5000, Bruker, Billerica, MA, USA: samples C3, C14, SU1, SU3; D8, Bruker, Billerica, MA, USA: samples C30, C32, SU24; raw data: see Supplementary Materials) in Bragg-Brentano geometry using Cu*K_α_* radiation. In the Siemens D5000 instrument, the goniometer caused a shift of +0.26° in the measured angles 2θ of the raw data. The respective data were corrected for graphical representation. Quantification of crystalline phases contained in the samples was achieved by refinement of the corresponding XRD patterns using the computer program Profex, version 3.11.1 [84].

### 3.4. Cation Release upon Dissolution

Sr-modified samples were investigated for the release of cations upon their reaction with Ringer’s solution, an aqueous solution containing NaCl (8.6 g/L), KCl (0.3 g/L), and CaCl_2_ · 2 H_2_O (0.33 g/L) at pH 7.4 (isotonic in relation to body fluids). Cleaved samples of about 50 mg were held in 20 mL of Ringer’s solution at 37 °C for 24 h and 72 h. A sample of pure Ringer’s solution served as a control. Resulting solutions were analyzed for Mg and Sr ions using flame atomic absorption spectroscopy (AAS; contrAA 300, Analytik Jena, Jena, Germany). Detection limits were 0.01 mg/L for Mg^2+^ and 0.08 mg/L for Sr^2+^. 

## 4. Conclusions

The presented study demonstrates the development of porous phosphatized Ca carbonate biominerals, specifically coral- and sea urchin-derived CaP scaffolds, into bioactive bone graft substitute materials with advanced functionality through the incorporation of Mg and Sr ions. The use of Mg-bearing sea urchin calcite as starting materials instead of the more commonly used coralline aragonite skeletons offered an effective way of introducing Mg ions to the hydrothermal mineral replacement reaction in a phosphate solution. These Mg ions shifted the equilibrium of the resulting CaP phases from nonresorbable HA toward merrillite (Na-Mg-β-TCP), which is resorbable as an implant. Upon resorption, the release of Mg ions is known to stimulate the formation of new bone tissue. An approach of EDTA-aided stabilization and transport of Sr ions in the hydrothermal phosphate solution allowed for the incorporation of these cations into the pseudomorphic CaP scaffolds as Sr-substituted β-TCP during the replacement of the original Ca carbonate material. Upon implantation and resorption of the materials, the release of Sr ions is expected to enhance the formation of dense bone tissue. In conclusion, the conventional coralline HA was improved by the incorporation of bioactive Sr ions, and equivalent sea urchin-derived CaP was developed as a promising alternative material. The sea urchin-derived material showed potential for better resorbability and performance than the coral-derived materials based on their different composition and additional release of Mg ions. With respect to potential adverse effects of residual organic matrices from the native biominerals, the sea urchin-derived material with very little organic content in the starting material seems to be the better choice. This fundamental study may be the basis for producing or developing new biomineral-derived implant materials as alternatives for CaP ceramics and bioglasses.

In future research on phosphatized Ca carbonate biominerals, the actual contents of residual organic substances should be analyzed, and the performance of these materials as implants remains to be tested in model systems. For further development, additional modifications of these materials could take them even further toward multifunctional implants. For example, in a second part of the study presented here, the materials discussed will be additionally functionalized with Ag ions for an antibacterial effect that may reduce the risk of perisurgical wound infections [85].

## Figures and Tables

**Table 1:** Ion concentrations determined using atomic absorption spectroscopy (AAS) after material dissolution in Ringer’s solution at 37 °C for 24 h and 72 h.

Sample	Reaction Time	Mg^2+^ (mg/L)	Sr^2+^ (mg/L)
C32	24 h	0.14	1.2
C32	72 h	0.1	1.3
SU24	24 h	0.16	0.31
SU24	72 h	0.19	0.32
Ringer	-	0.1	<0.08 ^1^

^1^ Detection limit.

## Supplementary Materials

The following are available online at http://www.mdpi.com/2079-4983/9/4/69/s1, Figure S1: Coral skeleton held in deionized water at 200 °C for 168 h; XRD raw data S1: C14; XRD raw data S2: SU1; XRD raw data S3: C30; XRD raw data S4: C3; XRD raw data S5: SU3; XRD raw data S6: C32; XRD raw data S7: SU24.
