# Supplementary material for: Development of Phosphatized Calcium Carbonate Biominerals as Bioactive Bone Graft Substitute Materials, Part I: Incorporation of Magnesium and Strontium Ions
Source: J Funct Biomater. 2018 Dec 2;9(4):69. doi: 10.3390/jfb9040069 (PMC6306735; doi:10.3390/jfb9040069)
Supplement: Supplementary file 1 [file jfb-09-00069-s001.zip › Figure S1.pdf]

Supplementary material

Development of phosphatized calcium carbonate biominerals as bioactive bone graft substitute materials, part I: incorporation of Mg and Sr ions

Ingo Sethmann [1,*], Cornelia Luft [1,2], Hans-Joachim Kleebe [1]

[1] Institute of Applied Geosciences, Technische Universität Darmstadt, 64287 Darmstadt, Germany

[2] Institute of Geosciences, Johannes-Gutenberg-Universität, 55128 Mainz, Germany

* Correspondence: ingo.sethmann@gmail.com

Coral skeleton held in deionized water at 200°C for 168 h.

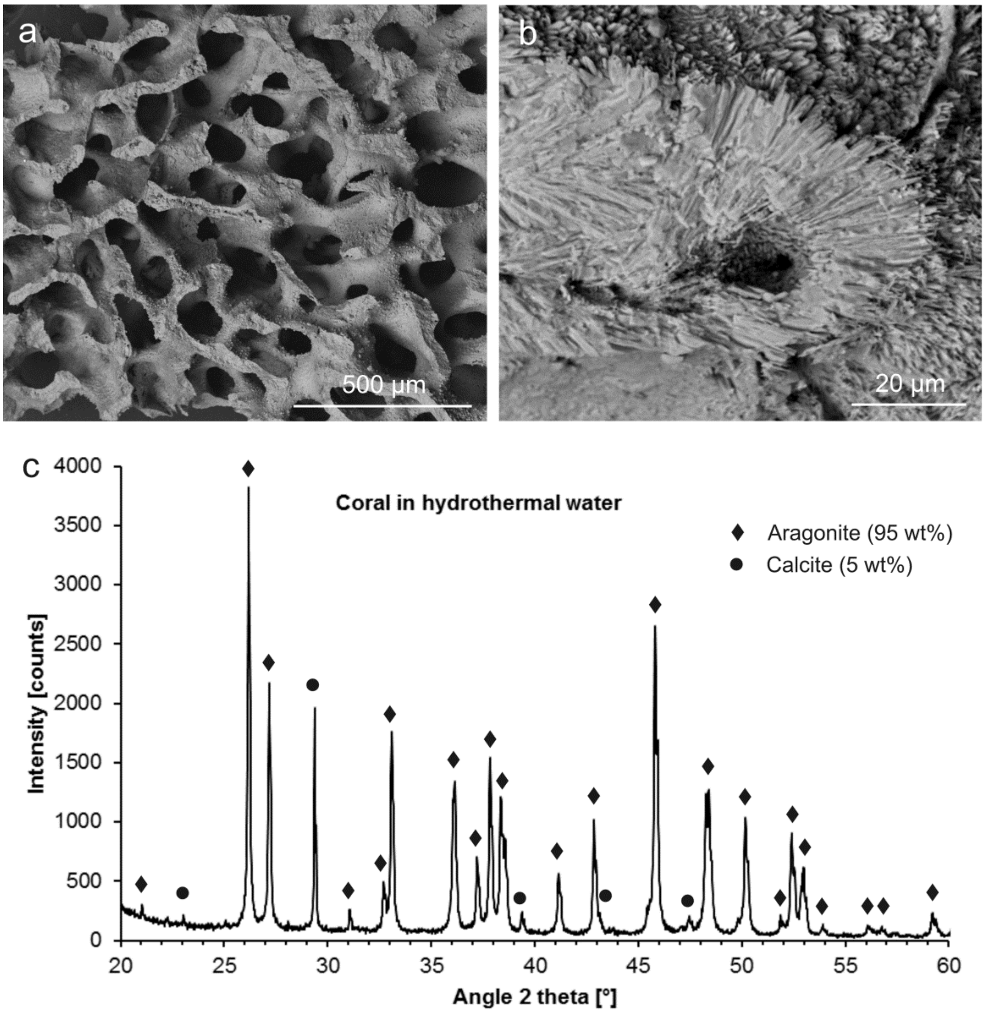

Figure S1. Coral skeleton held in deionized water at 200°C for 168 h. (**a**) Macromorphology and (**b**) internal microstructure of the material were preserved, while dissolution at the surface exposes the fibrous material; scanning electron microscopy (SEM), back-scattered electron images. (**c**) The hydrothermal treatment resulted in a partial replacement of the original aragonite (95 wt%; PDF 00-041-1475) by calcite (5 wt%; PDF 00-047-1743); X-ray diffractometry (XRD). XRD raw data S3: C30.
